# Prognostic Implications of Cutaneous Metastases in Colorectal Cancer: A Comprehensive Systematic Review and Meta‐Analysis

**DOI:** 10.1002/cam4.71197

**Published:** 2025-09-12

**Authors:** Elliot Tokarski, Pierre‐Louis Conan, Hugo Picchi, Brice Malgras, Evelyne Peroux, Anne‐Cecile Ezanno

**Affiliations:** ^1^ Department of Digestive Surgery Begin Military Teaching Hospital Saint Mandé France; ^2^ Department of Infectious Diseases Begin Military Teaching Hospital Saint Mandé France; ^3^ Department of Oncology Begin Military Teaching Hospital Saint Mandé France; ^4^ French Military Heath Service Academy, Ecole du Val De Grâce Paris France; ^5^ Deparment of radiology Begin Military Teaching Hospital Saint‐Mandé France; ^6^ INSERM, Univ Rennes, OSS (Oncogenesis, Stress, Signaling) Laboratory, UMR_S 1242 Rennes France

**Keywords:** chemotherapy, colorectal cancer, cutaneous metastases, skin metastases, surgery

## Abstract

**Background:**

Management of cutaneous metastases in colorectal cancer is crucial because it can significantly impact patient survival.

**Objective:**

To assess the global prognosis of skin metastases among patients treated for colorectal cancer.

**Methods:**

A systematic review of the PubMed database only was conducted for English articles or reports published between 1 January 1990 and 1 November 2023. Reports concerning clinical outcomes of patients with cutaneous metastases of colorectal cancer and systematic reviews of the literature were included. Study characteristics and results of eligible studies were independently extracted by two reviewers (A.C.E., E.T.). The histology, locations, and prognosis of patients with skin metastases of colorectal cancer were analyzed.

**Results:**

Data were obtained from almost 100 articles, most of which were case reports. Follow‐up data were available for 62 patients. The most common site was rectal adenocarcinoma (58%), followed by right and left colon adenocarcinomas. A significant number of these metastases were metachronous (61%). The median time to death was 5.5 months [interquartile range, 3–10], ranging from 1 to 60 months. Treatment of cutaneous metastases, even if associated with other metastases, significantly improved overall survival (*p* < 0.001; hazard ratio, 0.15), regardless of the proposed treatment.

**Conclusions:**

We carried out the most extensive review of the literature concerning cutaneous metastases of colorectal cancer to date. The review showed that cutaneous metastases are associated with a poor prognosis and short survival time. We suggest treating patients with cutaneous metastasis as if such metastasis were a surrogate marker or sentinel for aggressive metastatic disease.

## Graphical abstract

Patients with cutaneous metastases have a poor prognosis. Through a mechanism that is still poorly understood, the treatment of skin metastases may lead to improved survival in patients with metastatic colorectal cancer.

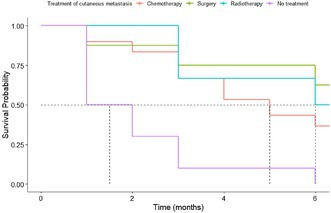

## 1 Introduction

Colorectal cancer is the third most prevalent cancer globally, accounting for approximately 1.9 million new cases annually. It is the second leading cause of cancer‐related mortality worldwide, with 930,000 deaths annualy [1]. Regardless of disease stage, the 5‐year survival rate is approximately 63%. Approximately 20% of patients present with metastasis at the time of diagnosis (synchronous), and 40%–60% are expected to develop metastases at some point during the disease course [2, 3].

The liver is the most common site for secondary metastases from colorectal cancer. Other less common sites include the lungs, peritoneum, and lymph nodes. The treatment recommendations often mimic those for hepatic disease, involving surgery or a combination of surgery and ablative treatments if feasible. Cutaneous metastases from colorectal cancer are rare, occurring in approximately 4% of cases [4, 5]. In most cases, colorectal cancer is diagnosed before the appearance of cutaneous metastases. The skin is an uncommon secondary site of metastases, accounting for 0.7%–9.0% of all cancers [6]. They may occur synchronously with the diagnosis of the primary disease, although it is rare for such metastases to be the initial indicator of colorectal cancer. Cutaneous metastases are often indicative of advanced disease and can pose diagnostic and therapeutic challenges. In certain scenarios, the primary diagnosis is established through the biopsy of a suspicious skin lesion [7].

Despite the numerous documented clinical cases of skin metastases from colorectal cancer, a comprehensive review of the literature summarizing and describing the characteristics of this type of metastasis has not yet been carried out. It is essential to explore the behavior of cutaneous metastases in colorectal cancer to establish an appropriate management strategy.

This study was performed to provide an overview of the clinical characteristics, diagnostic processes, and treatment options for cutaneous metastases in patients with colorectal cancer. We systematically reviewed all PubMed‐documented publications on cutaneous metastases from colorectal cancer to improve our understanding of how to manage this condition. By primarily analyzing case reports, we aimed to provide a comprehensive overview that highlights the characteristics and demographics of individuals with cutaneous metastases originating from colorectal cancer.

## 2 Methods

### 2.1 Search Strategy and Selection Criteria

This systematic review and meta‐analysis adhered to the Preferred Reporting Items for Systematic Reviews and Meta‐Analyses (PRISMA) guideline [4, 8]. The literature search was conducted in PubMed and focused on articles published between 1 January 1990 and 1 November 2023, using keywords and MeSH terms such as “cutaneous metastases,” “skin metastases,” “colorectal cancer,” “rectal cancer,” and “colon cancer.” Additional relevant studies were identified through previous literature reviews and reference screening. Titles and abstracts were initially screened, followed by a full‐text review of potentially relevant articles published in peer reviewed English‐language journals. Articles with inaccessible or inadequately reported data were excluded. The articles included were English‐language articles, systematic reviews, or case reports with our endpoint information present. We excluded articles not in English, or that did not present patient follow‐up, with missing data for the analysis.

### 2.2 Data Extraction and Quality Assessment

Two independent reviewers (E.T. and A.C.E.) extracted data on the study characteristics, patient demographics, tumor histology, metastatic details (number, location, synchronous or metachronous, interval between cancer diagnosis and cutaneous metastases appearance, specific treatments), and follow‐up data, with a particular focus on overall survival analysis when follow‐up data were available. Any discrepancies were resolved by consensus.

### 2.3 Statistical Analysis

For all descriptive analyses, continuous variables are presented as mean ± standard deviation or median and interquartile range [25th–75th percentile], and categorical variables are presented as number and percentage. For categorical variables, the chi‐square test was used to compare the two groups, adhering to the Cochran criteria [9]. If the Cochran criteria were not met, the nonparametric Fisher's exact test was used. Continuous parameters with a non‐normal distribution were analyzed using the Mann–Whitney test. Factors associated with death within 6 months post‐diagnosis were investigated by the Kaplan–Meier method and then by determination of hazard ratios (HRs) with 95% confidence intervals (CI) using a Cox proportional hazards model. In these survival analyses, time was defined as the duration from diagnosis to death. Variables with *p*‐values of < 0.2 in the univariate analysis, along with those found to be significant in the literature, were included in the multivariate analysis, with consideration for highly correlated variables. To test the proportional risks hypothesis, we inspected the nonparametric Kaplan–Meier curves for the qualitative variables and analyzed the temporal distribution of Schoenfeld residuals for the quantitative variables. A threshold of 5% was used to define the significance of the statistical tests. No adjustment for multiplicity was applied. All analyses were performed using R software version 4.4.0 (April, 2024).

## 3 Results

### 3.1 Study Selection and Characteristics

**FIGURE 1:**
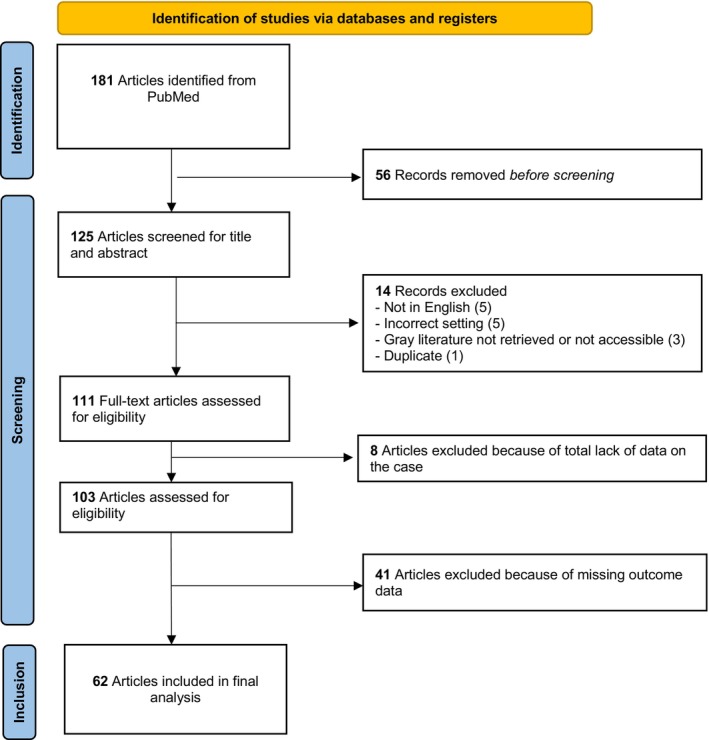
PRISMA 2020 flow diagram of new systematic reviews that included searches of databases.

In total, 181 articles were retrieved from PubMed. After initial screening based on title and abstracts, 125 articles were selected for further analysis. Fourteen articles were excluded because of insufficient data, and eight were excluded because of unavailable data. Upon full‐text review and reference list examination, 103 articles were ultimately selected for inclusion in this systematic review (Figure 1).

Among the 103 articles, 97 were case reports and 6 were reviews. The articles involved a total of 100 patients, among whom 62 were included in the Kaplan–Meier analysis. The study characteristics are summarized in Appendix S1. Data were collected for 100 patients with a mean age of 62 ± 14 years (range, 25–92 years). All patients' demographic and clinical characteristics are listed in Table 1. The study population included a higher proportion of men (59%) than women. Tumor localization was reported in 96 cases, with rectal adenocarcinoma being the most common (53%). This was followed by adenocarcinoma of the right colon and left colon, each accounting for 21% (*n* = 20/96) of the cases, and adenocarcinoma of the transverse colon, constituting 5.2% (*n* = 5/96).

**TABLE 1:** Demographic and clinical characteristics according to the analyzed population with follow‐up available.

Characteristic	Population included *n* = 100	Population excluded *n* = 38	Population analyzed *n* = 62	*p*
Age, years	62 (14) [51–71]	62 (14) [50–72]	62 (14) [52–71]	0.9
Sex				0.3
Female	41%	47% (18)	37% (23)	
Male	59%	53% (20)	63% (39)	
Tumor origin				0.11
Right colon	21% (20/96)	27% (10/37)	17% (10/59)	
Left colon	21% (20/96)	27% (10/37)	17% (10/59)	
Transverse colon	5.2% (5/96)	0% (0/37)	8.5% (5/59)	
Rectum	53% (51/96)	46% (17/37)	58% (34/59)	
Missing data			3	0.4
pN stage^a^				
pN0	24% (15/62)	18% (4/22)	28% (11/40)	
pN1/2	76% (47/62)	82% (18/22)	72% (29/40)	0.4
Metachronous cutaneous metastasis	59% (58/98)	56% (20/36)	61% (38/62)	0.6
Localization				0.4
Perineum	25% (25/100)	29% (11/38)	23% (14/62)	
Limb	9.0% (9/100)	11% (4/38)	8.1% (5/62)	
Thorax, abdomen, back	27% (27/100)	32% (12/38)	24% (15/62)	
Face	21% (21/100)	16% (6/38)	24% (15/62)	0.6
Several cutaneous metastases	17% (17/100)	11% (4/38)	21% (13/62)	
Other associated metastasis	**59% (42/71)**	**74% (20/27)**	**50% (22/44)**	**0.045**
Type of treatment for colorectal cancer				0.4
Chemotherapy	19% (15/77)	19% (5/27)	20% (10/50)	
Chemotherapy + surgery	27% (21/77)	19% (5/27)	32% (16/50)	
Surgery	47% (36/77)	56% (15/27)	42% (21/50)	
None	5.2% (4/77)	3.7% (1/27)	6.0% (3/50)	
Missing data	23	11	12	
Type of treatment for skin metastasis				
Chemotherapy	47% (40/85)	32% (10/31)	56% (30/54)	0.4
Surgery	24% (20/85)	39% (12/31)	15% (8/54)	
Radiotherapy	11% (9/85)	9.7% (3/31)	11% (6/54)	
None	16% (14/85)	13% (4/31)	19% (10/54)	
Missing data	15	7	8	
Interval between primary tumor and cutaneous metastasis	**19 (18) [6–30]**	**28 (22) [12–48]**	**15 (15) [5–22]**	**0.007**

*Note:* Data are presented as mean ± standard deviation, median [25th–75th percentile], or percentage (number). Bold values highlight the significant data of our analysis.

^a^ N stage according to the American Joint Committee on Cancer 7th edition.

Among the metastatic presentations, 59% (*n* = 58/98) were metachronous. Metastatic localizations varied, with the trunk being the most common site (27%), followed by the perineum (25%), face (21%), limbs (9%), multiple sites (17%), and other locations (1%). Additionally, 59% (*n* = 42/71) of the patients had metastases other than cutaneous ones. For those with metachronous lesions, the mean interval between the development of the primary tumor and the cutaneous metastasis was 19 ± 18 months (range, 1–72 months).

Follow‐up data were available for 62 patients (analyzed population). The demographic and clinical data for these two groups of patients are presented in Table 1. There were no significant differences between the groups in terms of age, sex, or localization. Primary treatment modalities showed no significant difference (*p* = 0.4), with surgery being the most common treatment in both groups.

**FIGURE 2:**
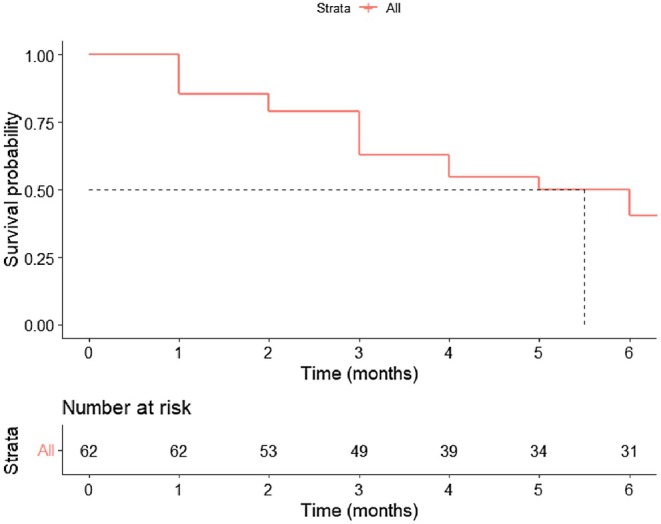
Kaplan–Meier overall survival curve. Kaplan–Meier survival curve showing the probability of survival over a 6‐month period.

Thirty‐seven of the 62 patients died during the 14,490 person‐days of follow‐up. The global incidence rate of death was 2.55 (95% CI, 1.73–3.38) per 1000 person‐days. The median time to death was 5.5 months (95% CI, 3–10; range, 1–60 months). The Kaplan–Meier overall survival curve is shown in Figure 2.

### 3.2 Factors Associated With Death (Univariate and Multivariate Analyses)

The univariate analysis identified age and pTNM stage of the primary tumor as factors significantly associated with a higher risk of death at 6 months (*p* = 0.037 and *p* = 0.011, respectively). The occurrence of multiple cutaneous metastases was significantly associated with a 2.51‐fold increase in the risk of death (HR = 2.51 [1.22–5.17], *p* = 0.012). Treatment of metastases was significantly associated with improved survival as indicated by the markedly reduced risk of death among treated patients (HR = 0.19 [0.09–0.41]).

In the multivariate analysis, the localization of the tumor was a significant factor. Left colon tumors were associated with an increased risk of death (HR = 2.87 [1.02–8.13]). The stage of the disease maintained its significance, with stage 4 having a higher adjusted HR (HR = 6.39 [1.37–29.9]), reinforcing that more advanced disease stages were associated with increased mortality. The treatment of metastasis remained highly significant for overall survival in the multivariate analysis (HR = 0.15 [0.05–0.44]), indicating that treatment of metastases was associated with lower death rates at 6 months. The presence of several cutaneous metastases was associated with lower overall survival (HR = 2.12 [0.81–5.55]) than with the presence of a single metastasis (Table 2).

**TABLE 2:** Univariate and multivariate analyses: Overall survival.

Factor	Univariate analysis		Multivariate analysis	
	Hazard ratio [95% CI]	*p*	Hazard ratio [95% CI]	*p*
Age	0.50 [0.26–0.97]	**0.037**	1.04 [0.46–2.37]	> 0.9
Sex				
Female	—			
Male	1.04 [0.53–2.04]	> 0.9		
Tumor origin		0.2		**0.038**
Rectum	—		—	
Right colon	0.43 [0.15–1.24]		0.35 [0.10–1.26]	
Left colon	1.17 [0.50–2.73]		2.87 [1.02–8.13]	
Transverse colon	0.53 [0.12–2.25]		0.48 [0.10–2.26]	
Metachronous skin metastasis	0.68 [0.35–1.30]	0.2	0.84 [0.32–2.26]	0.7
TNM stage		0.011		**0.042**
2	—			
3	2.37 [0.75–7.48]		3.16 [0.75–13.20]	
4	4.4 [1.47–13.20]		6.39 [1.37–29.90]	
Localization of skin metastasis		0.7		
Perineum, limb	—			
Thorax, abdomen, back	1.27 [0.51–3.12]	0.6		
Face	0.87 [0.32–2.34]	0.8		
Metastases other than cutaneous (yes)	1.41 [0.67–3.00]	0.4		
Primary treatment (yes)	0.50 [0.12–2.12]	0.3		
Metastasis treatment (yes)	0.19 [0.09–0.41]	**< 0.001**	0.15 [0.05–0.44]	**< 0.001**
Several cutaneous metastases (yes)	2.51 [1.22–5.17]	**0.012**	2.12 [0.81–5.55]	0.12

*Note:* A univariate Cox model was used. Bold values highlight the significant data of the analysis.

Abbreviation: CI, confidence interval.

**FIGURE 3:**
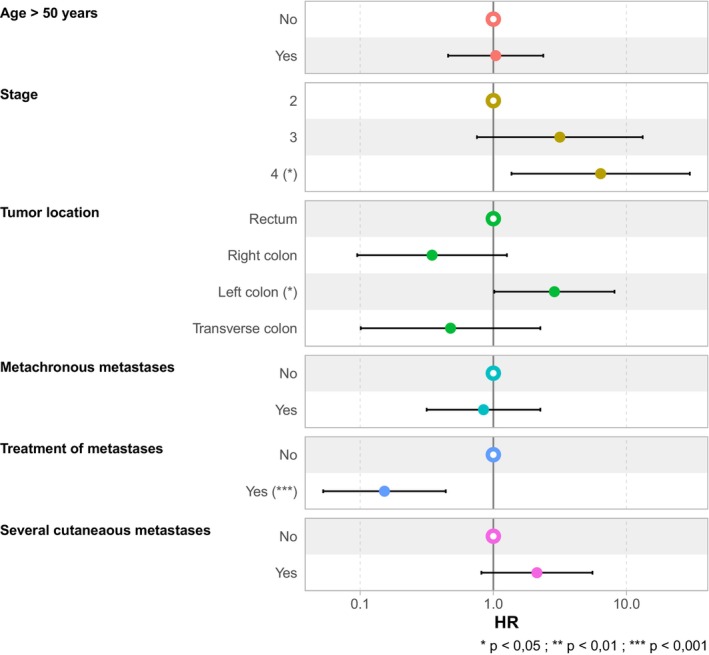
Forest‐plot of different factors associated with survival of patients with colorectal cancer with cutaneous metastases. Forest plot illustrating the HR for various factors. The plot includes age > 50 years, cancer stage, cancer localization, metachronous metastasis, metastasis treatment, and presence of several cutaneous metastases. Each factor is compared against a reference group, with hazard ratios and 95% confidence intervals displayed.

Figure 3 shows the results of the analysis including age, cancer stage, tumor localization, presence of metachronous metastasis, treatment of metastasis, and the occurrence of multiple cutaneous metastases. Significant associations are indicated, providing insights into the prognostic impact of these factors.

**FIGURE 4:**
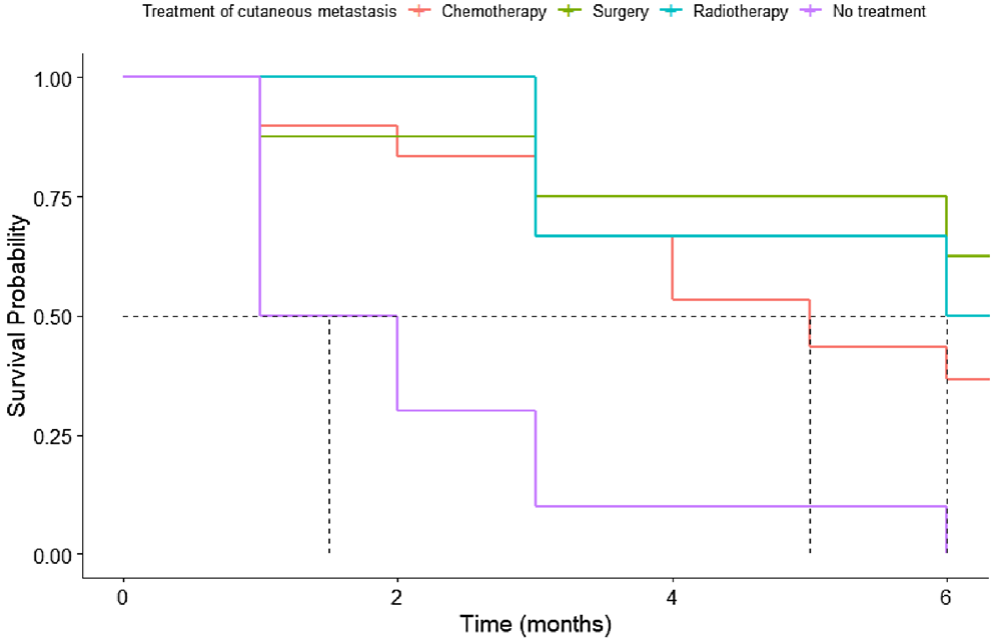
Kaplan–Meier overall survival curve according to cutaneous metastasis treatment. Kaplan–Meier survival curves comparing the probability of survival over a 6‐month period for patients with cutaneous metastasis receiving different treatments. The treatments include chemotherapy (red), surgery (green), radiotherapy (blue), and no treatment (purple). The plot shows survival probabilities decreasing over time, with distinct patterns for each treatment type. The dashed horizontal line at 0.50 indicates the median survival probability.

Patients who received treatment for cutaneous metastasis appeared to have a higher survival probability than those who received any treatment. Metastases may be treated with surgical resection, radiotherapy with or without surgery, or systemic chemotherapy. However, analysis of the type of cutaneous metastasis treatment showed that no treatment was superior to any other (Figure 4). Surgical removal of cutaneous metastases appeared to be associated with the best survival outcome among the treatments examined.

## 4 Discussion

Cutaneous metastasis of colorectal cancer is exceedingly rare, with an estimated incidence of 2.3%–6.0% [10]. This review, comprising data from 103 articles predominantly consisting of case reports, underscores the scarcity of documented cutaneous metastases and highlights the need for heightened clinical awareness and reporting. Our analysis revealed that patients with cutaneous metastasis from colorectal cancer have a poor prognosis, with a median survival time of < 6 months. Through a multivariate analysis, we identified factors associated with death. The primary factor associated with death within 6 months was the number of cutaneous metastases; survival significantly decreased when a patient had more than one cutaneous metastasis. Conversely, treatment of cutaneous metastases emerged as a factor associated with better survival, improving life expectancy regardless of the treatment approach (surgery, radiation therapy, or chemotherapy).

The management of cutaneous metastases from colorectal cancer remains uncertain in the literature. Because of the rarity of these metastases and the associated challenges in conducting studies, specific treatment guidelines for these metastases are not addressed in French [11], European, or American guidelines [12]. If we consider other cancers causing cutaneous metastases, the literature and the management recommendations are almost nonexistent. Breast cancer is the most common etiology of cutaneous metastases in women, with 5%–10% [13, 14]. The causes are more varied in men, with lung cancer accounting for 24%, colorectal cancer for 19%, melanoma for 13%, and head and neck cancers for 12% [15]. International guidelines lack a unified approach for managing cutaneous metastases, regardless of the cancer type. While European guidelines for breast cancer suggest systemic treatments along with local options like surgery, radiation, or ablation, they fall short of specifically addressing cutaneous metastases. Similarly, there are no definitive recommendations for treating cutaneous metastases from lung or head and neck cancers [16, 17, 18].

Treatment for a single skin lesion typically involves surgery, radiation therapy, or a combination of chemotherapy and surgery. When multiple lesions are present, chemotherapy alone may be considered; however, it can sometimes result in an insufficient response due to a reduced blood supply to the skin. When the lesions are bleeding or causing pain, palliative radiation therapy may be employed to alleviate symptoms.

For patients diagnosed with synchronous metastases from colorectal cancer, radical treatment of metastases may improve clinical outcomes and help achieve sustained cancer control or remission. French and American guidelines agree on the treatment approach to resectable hepatic and extrahepatic metastases, emphasizing resectability and operability, a performance status of 0 or 1, R0 resection without vascular invasion (i.e., complete removal of the tumor with no microscopic residual tumor lesion), and a residual liver volume of > 25% [10, 11]. If the metastases are resectable, the protocol involves presurgical chemotherapy, evaluation after four to six cycles, and subsequent hepatic resection and postoperative chemotherapy cycles. Depending on the case, the decision to perform a resection of the colonic tumor may vary. For resectable extrahepatic metastases, such as those in the lung, the approach mirrors that for the liver, favoring surgery if complete resection is possible [11]. For cerebral or adrenal metastases, the complete resection of which is not feasible, local treatments such as radiofrequency ablation or radiation therapy are preferred. For metastases in the lungs, peritoneum, or lymph nodes, recommendations tend to mimic those for hepatic disease, advocating for surgery or a combination of surgery and ablative treatments if possible [11]. The management of cutaneous metastases stands out as a critical factor in patient survival. Our findings corroborate the notion that active treatment, whether local, systemic, or a combination thereof, is associated with improved outcomes. This is evident from our survival analysis, which showed that untreated patients had markedly poorer prognoses. It is also important to note the classic observation that, in retrospective analyses, untreated patients are often those with the most severe disease.

We therefore propose that, wherever possible, treatment of these metastases should be considered as soon as the patient's condition allows, without however being able to recommend one type of treatment over another. Treatment must be carried out even if the patient has metastatic localizations other than the skin because such patients do not have a significantly poorer outcome. Our multivariate analysis identified the multiple number of cutaneous metastases as a primary factor associated with decreased survival, highlighting the importance of early detection and intervention.

### 4.1 Limitations

This study had several limitations. First, only case reports were analyzed. This introduces selection bias because only rare cases are published, in contrast to a systematic analysis of a cohort or registry of metastatic colorectal cancer. Second, the follow‐up time was short. Third, comprehensive data were lacking, preventing direct comparison of different treatments. Fourth, publication bias is a concern because positive results are often overrepresented, leading to an overestimation of favorable outcomes. Fifth, our meta‐analysis did not systematically evaluate biases and the quality of the case reports. Finally, the study was limited to data from PubMed. A more comprehensive analysis might have been possible with registry data; however, the largest study to date included only 55 cases and was limited to a descriptive literature review without a univariate or multivariate statistical analysis [18]. Despite these limitations, we were able to conduct a multivariate analysis and demonstrate an increase in survival in treated patients, indicating that multimodal treatment plus management of cutaneous metastases can improve patient survival.

## 5 Conclusion

Our systematic review highlights that cutaneous metastasis in colorectal cancer, particularly when multiple, is associated with an extremely poor prognosis, worse than that of metastatic disease to other organs. Despite their rarity, their presence marks a turning point in disease progression, with significantly reduced survival outcomes. However, our findings also reveal that patients who receive treatment for cutaneous metastases have better survival, not necessarily due to a reduction in tumor burden, but possibly through improved systemic disease control or supportive care. These results expose a critical gap in current guidelines, emphasizing the urgent need for dedicated treatment protocols. Larger, registry‐based studies are essential to confirm these observations and optimize management strategies to improve patient outcomes.

## Author Contributions

**Elliot Tokarski:** conceptualization (equal), methodology (equal), writing – original draft (equal). **Pierre‐Louis Conan:** conceptualization (equal), formal analysis (equal), methodology (equal), writing – review and editing (equal). **Hugo Picchi:** writing – review and editing (equal). **Brice Malgras:** supervision (equal), writing – review and editing (equal). **Evelyne Peroux:** writing – review and editing (equal). **Anne‐Cecile Ezanno:** conceptualization (equal), formal analysis (equal), methodology (equal), validation (equal), writing – review and editing (equal).

## Conflicts of Interest

The authors declare no conflicts of interest.

## Supporting information


**Appendix S1:** cam471197‐sup‐0001‐AppendixS1.xlsx.

## Data Availability

A detailed description of the articles reviewed is available in Table S1 in Appendix S1. Other data are available for *bona fide* researchers on written request to the authors.
